# Milestones achieved in response to drought stress through reverse genetic approaches

**DOI:** 10.12688/f1000research.15606.1

**Published:** 2018-08-17

**Authors:** Baljeet Singh, Sarvjeet Kukreja, Umesh Goutam

**Affiliations:** 1 Biotechnology, Lovely Professional University, Phagwara, Punjab, 144411, India; 2 Department of Botany, Ch. MRM Memorial College, Sriganganagar, Rajasthan, 335804, India

**Keywords:** VIGS, CRISPR, TILLING, ESTs, Drought stress, Climate change, Reverse Genetics, Functional genomics

## Abstract

Drought stress is the most important abiotic stress that constrains crop production and reduces yield drastically. The germplasm of most of the cultivated crops possesses numerous unknown drought stress tolerant genes. Moreover, there are many reports suggesting that the wild species of most of the modern cultivars have abiotic stress tolerant genes. Due to climate change and population booms, food security has become a global issue. To develop drought tolerant crop varieties knowledge of various genes involved in drought stress is required. Different reverse genetic approaches such as virus-induced gene silencing (VIGS), clustered regularly interspace short palindromic repeat (CRISPR), targeting induced local lesions in genomes (TILLING) and expressed sequence tags (ESTs) have been used extensively to study the functionality of different genes involved in response to drought stress. In this review, we described the contributions of different techniques of functional genomics in the study of drought tolerant genes.

## Introduction

Nowadays, global food security has becomes a major challenge due to the extreme changes to the climate and increases in the global population ^1^. Therefore, plants are growing under various kinds of unfavourable environmental stresses such as drought, salinity, heat, cold and oxidative stresses which are retarding the growth and yield ^2, 3^. Of these, drought stress is the most predominant abiotic stress making this situation worse. Over the last decade, climate change has been increasing the frequency drought conditions and reduced the crop yield ( Table 1) by affecting the basic plant growth processes such as seed germination, photosynthesis, source sink relationships, turgor pressure, cell division and elongation, enzyme activities, and secondary metabolites production ^14– 24^. In addition, drought can also increase the production and accumulation of reactive oxygen species (ROS) in plants which leads to oxidative stress too ^25, 26^. Several genes that express under drought conditions are involved in the regulation of all these processes and pathways. In recent years, many drought tolerant genes have been identified in major food crops and still there are numerous genes taking part in drought stress whose functions are unknown. With the help of available genomic and transcriptomic data reverse genetic approaches accelerated the investigations of gene function under different abiotic stresses ^27^.

**Table 1.** Yield loss in various crops under different drought conditions.

Crop	Yield loss (approx. %)	References
Wheat	57 21 26	4 5 6
Rice	53–92	7
Maize	63–87 40 40	8 5 6
Chickpea	45–69	9
Soybean	46–71	10
Sunflower	60	11
Potato	17	12
Barley	37–41	13

From the perspective of crop improvement, transgenic approaches have been successfully used in many crops. However, development of stable transgenic lines is relatively expensive, time consuming and a laborious task. Moreover, it is not successful in many cultivated crops and slows down the investigations into specific gene ^28^. In contrast, there are several techniques available for the study of these genes which give prompt results and have other advantages over transgenic techniques for analysis of target gene(s) such as virus-induced gene silencing (VIGS), clustered regularly interspace short palindromic repeat (CRISPR)-Cas9 system, targeting induced local lesions in genomes (TILLING) and expressed sequence tags (ESTs) ^29– 32^.

## VIGS

It is a simple, rapid, reliable and cost-effective post-transcriptional gene silencing (PTGS) technique for the study of endogenous genes. It is a powerful tool for the mining and study of genes involved in drought tolerance ( Table 2). In VIGS a 200-400bp long fragment of the target gene is selected and cloned into a viral vector which infects the plant and triggers the silencing of that particular gene ^29, 55^. For efficient gene silencing the selection of the target fragment is very crucial. This technology can be used for forward and reverse genetics for both monocotyledonous and dicotyledonous plants ^56, 57^. There is no requirement of stable plant transformants in VIGS technology ^58^. Moreover, a number of different genes can be studied simultaneously and a specific target can also be silenced individually through this technology ^59, 60^. Many VIGS vectors have been developed for different crops by modifying plant viruses and have been used successfully for the functional study of genes expressed under drought stress ^61– 64^. These VIGS vectors along with the target gene can be inoculated into the plants by different methods such as agrodrench, needleless syringe inoculation, agro inoculation, prick inoculation, and biolistic inoculation ^29, 65^.

**Table 2.** Successfully confirmed genes via different virus-induced gene silencing (VIGS) systems.

Viral vector	Type	Crop	Target gene	Reference
BSMV (Barley Stripe Mosaic Virus)	RNA virus	Wheat Barley	*TaEra1,* *TaSal1* *TaBTF3* *TaPGR5* *TdAtg8* *HvHVA1* *HvDhn6* *HvEXPB7* *HvATG6*	33 33 34 35 36 37 37 38 39
TRV (Tobacco Rattle Virus)	RNA virus	Tomato *Pyrus betulaefolia* Chili pepper Cotton	*Sllea4* *SpMPK1,* *SpMPK2,* *SpMPK3* *SlMPK4* *SlSR1L* *SlJUB1* *PbrMYB21* *CaPO2* *CaMLO2* *CaAIR1* *CaAIP1* *CaWDP1* *GhMKK3* *GhWRKY27a*	40 41 41 41 42 43 44 45 46 47 48 49 50 51 52
TYLCCNV (Tomato Yellow Leaf Curl China Virus)	DNA virus	Tomato	*SlGRX1*	53
CLCrV(Cotton Leaf Crumple Virus)	DNA virus	Cotton	*GhNAC79*	54

Plants have adopted many molecular mechanisms to withstand different abiotic stress, and a number of stress related genes get stimulated under stress conditions ^66, 67^. Among them, MAPKs (Mitogen Activated Protein Kinases) are the most important enzymes for the plant growth and development and also play an important role in signal transduction under extreme conditions ^68– 72^. The role of different MAPKs under drought stress has been studied through VIGS technology. The silencing of genes *SpMAPK1*, *SpMAPK2*, *SpMAPK3* in *Solanum pimpinellifolium, SlMPK* in *Solanum Lycopersicum* and *GhMKK3* in *Gossypium hirsutum* reduced the drought tolerance in silenced plants ^41, 42, 51^.

In addition, various transcriptional factors regulate the plants behaviour in response to environmental conditions ^73^. The WRKYs transcription factors play crucial role in the plant development under drought stress ^74^. In cotton the VIGS of *GhWRKY27a* enhanced the tolerance against drought stress ^52^. Further, another family of transcriptional factor, NAC, plays an important role under drought ^75^. Silencing of the *GhNAC79* and *JUB1* genes in cotton and tomato respectively made the plants more sensitive to drought ^44, 54^. In addition, *PbrMYB21* gene belonging to MYB family of transcription factors (TFs) studied in *Pyrus betulaefolia.* The *PbrMYB21* silenced plants exhibited decreased drought tolerance in comparison to control plants ^45^. Beside these, SR/CAMTA proteins from a small family of TFs and silencing of *SISR1L* and *SlGRX1* genes from this resulted in decreased tolerance against drought stress in tomato ^43, 53^. 

Beside these, autophagy, a protein degradation process induced in plants in response to environmental stimuli, has been reported to be involved due to the involvement of autophagy-related genes (ATG) under drought stress ^76– 78^. The *ATG8* gene in wheat and *ATG6* and its orthologs get induced in wheat, rice and barley in response to multiple abiotic stresses. Barley stripe mosaic virus (BSMV) based VIGS system was used for their functionality under drought stress. The results indicated the active participation of *ATG* genes in various survival mechanisms used by plants under drought ^36, 39^. In spite of these, many drought tolerant genes have been reported in weeds and also wild species of major cultivars. For instance, *ApDRI15* gene in a weed named, *Alternanthera philoxeroidsi* has been identified as a drought tolerant gene through VIGS ^79^.

## Expressed sequence tags (ESTs)

It is a sequence based technique that can be used to identify or study genes. ESTs can be generated from cDNA libraries ^80^. Functional studies of specific genes using this technique, can provide results in a cost-effective manner ^81^. Large scale EST sequencing has been performed in various crops and in several crops it is in progress. Millions of ESTs of different crops are available at Expressed Sequence Tags Database of National Center of Biotechnology Information. To identify drought stress responsive genes, first cDNA libraries are developed from plants growing under stressed conditions or from drought challenged tissues of drought responsive genotypes. Then by sequencing the clones ESTs can be identified ^80, 82^. ESTs provide high quality transcripts for investigation of genes as functional markers under stress conditions. During the last two decades, drought responsive genes have been identified and studied by ESTs in a number of crops such as common beans ^83^, barley ^84^, chickpea ^85– 88^, sorghum ^89, 90^, rice ^91– 94^, *Camelina sativa* ^95^, wheat ^96, 97^ Kodo millet ^98^, pearl millet ^99, 100^, sweet potato ^101^, rapeseed ^102^, Peanut ^103^, and *Ammopiptanthus mongolicus* ^104^. Analysis by BLASTX or qRT-PCR can be performed to find the most promising ESTs ^82^. 

## TILLING

With the advancements in high-throughput techniques genomes of a large number of crops are available now which present a number of new opportunities for the application of traditional mutation based reverse genetic techniques ^105^. TILLING is a nontransgenic method used to study allelic variations in the target gene in a mutant population and the effect of the mutant gene is studied from the changes in plant phenotypes ^28, 106, 107^. It is a quick and comparatively cheap method for the screening of single nucleotide polymorphisms (SNP) in the target sequence. These point mutations in the target genes can be identified by PCR ^105, 108^. Moreover, this technique is applicable to any plant species whose genome sequence is available, regardless of its ploidy levels. In TILLING, to induce mutations in plant genome chemical mutagens are used that generated random mutations ^105^. In most of the experiments, to generate the TILLING population ethymethansulfonate (EMS) is used as a mutagen ^30^. However, to study the polymorphism developed, due to environmental conditions, a modified technique called as EcoTILLING has been developed. It seems a more promising strategy to study the genes related to abiotic stresses ^109, 110^.

## CRISPR Technology

CRISPR (Clustered Regularly Interspace Short Palindromic Repeat)/ CRISPR-associated nuclease protein (Cas) 9 technology based upon plant antiviral defense mechanisms, offered various new opportunities for researchers. It is relatively simple, easy, less cytotoxic and very efficient targeted genome editing technology in comparison to traditional techniques used for the same purpose ^111, 112^. CRISPR/CAS9 based gene editing technology has become common practice in various labs. It involves the use of the CAS9 endonuclease, originally derived from *Streptococcus pyogenes,* and a guide RNA which leads CAS9 to the target sequence working together and generate double stranded DNA breaks which are later repaired by the error prone non-homologous end joining (NHEJ) method or by the homology directed repair (HDR) pathway ^113, 114^. Recently, this technology has been used extensively for crop improvement ^31, 115– 120^. This system has been successfully used to study the genes involved in drought stress ( Table 3) in model plant Arabidopsis ^121^ and also in a number of crops such as soybean, maize ^114, 122^, rice ^123^, tomato ^124^.

**Table 3.** Recent examples of drought associated genes studied by clustered regularly interspace short palindromic repeat (CRISPR)/Cas9.

Sr. No.	Crop	Gene Name	Reference
1.	Arabidopsis	*mir169a*	113
2.	Arabidopsis	*UGT79B2,UGT79B3*	121
3.	Maize	*ARGOS8*	114
4.	Tomato	*slmapk3*	124
5.	Arabidopsis, Poplar	*PtoMYB216*	125
6.	Rice	*OsSAPK2*	123

## Conclusion and future perspectives

Severe droughts are becoming more common every year and are reducing crop yield considerably. There is an urgent need for drought tolerant varieties. Breeding and transgenic approaches could solve this problem but the knowledge of molecular mechanisms and genes taking part in drought tolerance is essential. Several reverse genetic techniques have proved their potential in many crops and some are still evolving. During the last decade, the genomes of several crops were successfully sequenced, various new VIGS systems have been developed for different crops ^104– 131^ and CRISPR has become the most powerful tool for genome editing ^126– 131^. Thus, these techniques can play a pivotal role in crop improvement and can contribute highly in the development of drought tolerant varieties.

## Data availability

No data are associated with this article
