# Supplementary material for: Milestones achieved in response to drought stress through reverse genetic approaches
Source: F1000Res. 2018 Aug 17;7:1311. [Version 1] doi: 10.12688/f1000research.15606.1 (PMC6290974; doi:10.12688/f1000research.15606.1)
Supplement: Peer review history

# Peer review: Referee response for version 1

**DOI:** 10.5256/f1000research.17024.r40635

**Published:** 2018-12-10

**Authors:** Pardeep Kumar Bhardwaj

**Affiliations:** 1 Institute of Bioresources and Sustainable Development, Sikkim Centre, Gangtok, Sikkim, India

The review entitled “Milestones achieved in response to drought stress through reverse genetic approaches” by Singh *et al.,* presents recent advances in understanding the response of drought stress in crop plants using reverse genetic technologies. It is well known that drought stress is major concern in the era of climate change. Therefore, it is important to address the recent updates on drought stress response in crop plants to scientific communities. The review article is written and organized very well but minor points need to be taken care.

1. Authors have explained several techniques available to study the functionality of different genes involved in response to drought stress but should also include the advantages of these techniques in monocots/dicots.
2. In VIGS, authors should explain the functional analysis of *DREB* transcription factors using VIGS technology citing some latest references.
3. In ESTs analysis, authors should include the analysis of drought responsive ESTs generated through chemical priming studied in crop plants.

I have read this submission. I believe that I have an appropriate level of expertise to confirm that it is of an acceptable scientific standard.

# Peer review: Referee response for version 1

**DOI:** 10.5256/f1000research.17024.r38198

**Published:** 2018-10-31

**Authors:** Elangovan Mani

**Affiliations:** 1 Advanta India Ltd., Hyderabad, Telangana, India

**Title:** Ok, it represents the article well 

**Abstract: **Ok, it provide the central idea about the article precisely. However, authors can expand it a little more.

**Keywords:**  Authors should arrange them alphabetically

**Introduction:** At the end of first paragraph, authors suggested about available transcriptomic data. I believe there they can add some references of available transcriptomes related to the topic. Overall, it has been written fine.

**VIGS: **Authors provided all the required information related to this technology. However, there are some bioinformatics tools, which helps in the selection of target fragment within the gene. They can add these too.

**Expressed sequence tags (ESTs):**  Ok

**TILLING: **Ok

**CRISPR Technology: **Ok

**Conclusion: **Ok, Nicely written.

 To summarize, the review by *Singh et al. *presents our current knowledge of many genes deciphered by reverse genetic technologies: *VIGS, EST, TILLING *and *CRISPR. *The tables represents ample amount of information in a well-organised way. Overall, the Review provides a useful compilation of subject matter related to addressed topic in a coherent way.

I have read this submission. I believe that I have an appropriate level of expertise to confirm that it is of an acceptable scientific standard.
